# Age effects of Moso bamboo on leaf isoprene emission characteristics

**DOI:** 10.3389/fpls.2023.1132717

**Published:** 2023-03-07

**Authors:** Yandong Song, Chunju Peng, Qinjiao Wu, Shijie Tao, Tingting Mei, Zhihong Sun, Zhaojiang Zuo, Chunyu Pan, Yufeng Zhou, Guomo Zhou

**Affiliations:** ^1^ State Key Laboratory of Subtropical Silviculture, Zhejiang A&F University, Hangzhou, China; ^2^ Lishui Academy of Agricultural and Forestry Sciences, Lishui, China; ^3^ Wenzhou Vocational College of Science and Technology, Wenzhou, China; ^4^ Key Laboratory of Carbon Cycling in Forest Ecosystems and Carbon Sequestration of Zhejiang Province, Zhejiang A&F University, Hangzhou, China; ^5^ College of Horticulture Science, Zhejiang A&F University, Hangzhou, China; ^6^ Zhejiang Provincial Key Laboratory of Forest Aromatic Plants-based Healthcare Functions, Zhejiang A&F University, Hangzhou, China; ^7^ Faculty of Forestry, University of British Columbia, Vancouver, BC, Canada

**Keywords:** Moso bamboo, isoprene, photosynthesis, light dependency, temperature dependency, G93 algorithm

## Abstract

Isoprene is a highly reactive volatile organic compound that significantly affects atmospheric oxidant capacity, regional air quality, and climate change. Moso bamboo (*Phyllostachys edulis*), a species widely distributed in tropical and subtropical regions, particularly in China, is a strong isoprene emitter with great potential for carbon sequestration. Carbon sequestration is negatively correlated with culm age; however, the effect of this correlation on isoprene emissions remains unknown. In this study, we investigated the photosynthetic and isoprene emission characteristics of Moso bamboo at different culm ages. The results showed that the age effect on isoprene emission was different from that on photosynthesis; the net photosynthesis rate (Pn) was the highest in young, followed by mature, and then old bamboo, whereas the isoprene emission rate (Iso) was the highest in young, followed by old, and then mature bamboo. Moreover, the percentage of carbon loss as isoprene emission (C-loss) during photosynthesis of old bamboo was 35% higher than that of mature bamboo under standard conditions (leaf temperature: 30°C; light intensity: 1000 µmol m^-2^ s^-1^). Therefore, we strongly recommend considering the culm age when establishing an isoprene emission model of Moso bamboo. Additionally, because the Iso and C-loss of old bamboo were higher than those of mature bamboo, we suggest that attention should be paid to the management of bamboo age structure and timely felling of aged bamboo to reduce environmental risk.

## 1 Introduction

Terrestrial vegetation releases massive volumes of biogenic volatile organic compounds (BVOCs) to the atmosphere at a rate of approximately 1 Pg C annually on a global scale (Guenther et al., 2012). These substances influence oxidants and aerosols, resulting in complex feedback on air quality and climate (Paulot et al., 2009). Isoprene (C_5_H_8_) is the dominant BVOC emitted into the atmosphere and constitutes approximately half of the total global BVOCs (Guenther, 2008; Guenther et al., 2012). Owing to its high abundance and atmospheric reactivity, isoprene plays a crucial role in tropospheric chemistry and climate change, particularly in the production of ozone (O_3_) and secondary organic aerosols (Fehsenfeld et al., 1992; Claeys et al., 2004; Mentel et al., 2013). Furthermore, isoprene competes with methane for radicals, such as hydroxyl, subsequently resulting in a longer lifetime of methane in the atmosphere and intensifying the global greenhouse effect (Poisson et al., 2000; Guenther, 2008). Therefore, the quantitative prediction of plant isoprene emissions is essential for understanding climate change and assessing air quality at the regional scale (Guenther et al., 2006; Monson et al., 2012). However, isoprene emissions are species-dependent and strongly influenced by environmental conditions (Sharkey et al., 1996; Harley et al., 1999; Lyu et al., 2021); most studies primarily focus on arbor species with high emissions (e.g., Populus, Eucalyptus, Quercus).

Bamboos are considered a novel system for studying BVOC emissions because their emissions are widely variable across species (Melnychenko and Rosenstiel., 2015). Moso bamboo (*Phyllostachys edulis*) is a strong isoprene emitter (Okumura et al., 2018; Chang et al., 2019), and it is expected to be a model plant of the grass family Gramineae to study isoprene emission (Melnychenko and Rosenstiel., 2015). Moreover, Moso bamboo has a wide distribution and a large planting area. It is widespread in the tropical and subtropical areas of east and southeast Asia, particularly China, as 4.43 Mha of Moso bamboo forest accounts for 84.02% of the species’ global range (Song et al., 2017). Additionally, its distribution area has been rapidly expanding owing to global climate change (Song et al., 2011). Owing to their monoculture, bamboo forests account for 97% of the total isoprene emissions in some rural forests (Guo et al., 2013).

Although the release of isoprene from Moso bamboo may have negative effects on the environment, the plant has many benefits. Moso bamboo is an excellent alternative to wood and has widespread applications in landscaping, industry, and daily life, significantly contributing to local economic development (Yin et al., 2019; Wang et al., 2021). Moso bamboo has tremendous potential for carbon (C) sequestration and climate change mitigation thanks to its rapid growth and robust regenerative capacity (Zhou et al., 2011; Song et al., 2017). Additionally, as a typical clonal plant from the Gramineae family, Moso bamboo forests consist of culms of varying ages developing in conjunction with a network of interlinked rhizomes. To maintain the sustainability and productivity of the stands, culms are harvested at five years of age (Yen and Lee, 2011; Zhou et al., 2011; Yen, 2017). Owing to a lack of secondary development and the blocking of xylem vessels by tyloses, aged culms are particularly susceptible to xylem dysfunction; this can lead to a progressive loss of photosynthates, nutrients, and conductivity for water and, eventually, culm mortality (Liese and Weiner, 1996). Moreover, leaf morphology (Hu et al., 2021) and photosynthesis (Kleinhenz and Midmore, 2001; Peng et al., 2021) depend not only on the leaf age but also on the culm age. However, whether culm age affects leaf isoprene emissions is unclear.

Isoprene biosynthesis is closely related to photosynthesis. Therefore, in addition to focusing on the potential negative impact of isoprene emissions on the environment, the net photosynthetic productivity of forests should be considered. Isoprene is primarily produced from newly absorbed photosynthates *via* the 2-C-methyl-D-erythritol 4-phosphate (MEP) pathway under light (Niinemets and Sun, 2015; Pokhilko et al., 2015). Under normal conditions, the majority (70–90%) of isoprene formations is directly from photosynthesis, which provides carbon skeletons, reducing power (NADPH), and energy (ATP) for isoprene biosynthesis (Delwiche and Sharkey, 1993; Karl et al., 2002; Affek and Yakir, 2003; Loreto et al., 2004). Therefore, photosynthesis and isoprene emissions increase with the increasing light intensity. When photosynthesis reaches its light saturation point, excess ATP and NADPH continue to be consumed by the MEP pathway, which lead to a higher light saturation point of isoprene (Wang et al., 2022). However, under environmental conditions that limit photosynthesis, alternative carbon sources that have not been recently assimilated can be mobilized for isoprene synthesis (Karl et al., 2002; Brilli et al., 2007; Jud et al., 2016). This mobilization leads to plant carbon loss because the carbon captured by photosynthesis is released back into the atmosphere as isoprene. The proportion of carbon re-emitted as isoprene typically accounts for 1% to 3% of the net primary output (de Souza et al., 2018). However, under adverse climatic conditions, this percentage can considerably increase to 50% (Sharkey and Loreto, 1993; Harley et al., 1999; Jansson et al., 2021). One example is the difference in the sensitivity of isoprene and photosynthesis response to temperature. The optimum temperature of isoprene is usually higher than that of photosynthesis, which leads to a decrease in photosynthesis with increasing temperature, while isoprene emission continues to increase when the temperature exceeds the optimum temperature for photosynthesis (Taylor et al., 2019; Rodrigues et al., 2020). However, the relationship between isoprene emissions and photosynthesis under different levels of environmental stress is not well understood for Moso bamboo.

Modeling is an efficient tool to quantify isoprene emissions at both stand and regional scales. The model of the G93 algorithm (Guenther et al., 1993), which includes the parameter of the basal emission factor (Es) and functions dependent on light and temperature, has been commonly used to evaluate isoprene emission. Es must be determined from the emission datasets measured by specific species; however, some empirical coefficients relating to light and temperature may need to be modified owing to species- and environment-specific variations (Alves et al., 2014; Okumura et al., 2018; Oku et al., 2021). Although a few studies have quantified isoprene emission fluxes from Moso bamboo leaves, most of these studies have only examined isoprene emission for a limited number of research samples (e.g., three leaves) over a short period (a few hours or a day) with limited temperature changes (no high temperature involved); moreover, culm age was not considered.

In this study, we synchronously measured the isoprene emissions and photosynthetic characteristics of Moso bamboo at different culm ages to analyze their age effects. Subsequently, in terms of carbon budget, we computed the ratio of carbon released as isoprene to carbon fixed by photosynthesis to evaluate the environmental cost in the process of carbon sequestration. Additionally, we determined the basal emission rate of isoprene and its response function with light and temperature, for providing basic data for the establishment of an isoprene estimation model, and evaluated the applicability of the G93 algorithm in bamboo.

## 2 Study site and materials

### 2.1 Site description and bamboo stand selection

Experiments were conducted at Lin’an (30°14’ N, 119°42’ E), Hangzhou City, Zhejiang Province, China, which is in a subtropical humid climate zone with an average annual temperature of 17.0 ± 2.6°C and an average annual precipitation of 1522.6 ± 518.8 mm (Tong et al., 2021). The Moso bamboo woods of the study site naturally regenerate without rigorous management and are positioned on the north slope of a low hill with a slope gradient of 36.5°. In this standing, the stand density was 1943 culms ha^-1^, with the young (1 year old), mature (1–5 years old), and old (≥6 years old) making up around 35%, 30%, and 35%, respectively, of the total density. The average height was 12.1 ± 3.1 m, and the average diameter at breast height (DBH) was 9.5 ± 1.7 cm.

The growth habit of Moso bamboo is markedly different from other arborous tree species. Most shoots sprout from the ground at the end of March, growing to their full height and DBH within the next two months (Song et al., 2016; Mei et al., 2020). The leaves begin to grow rapidly after the stands reach their maximum heights; in the following spring, they all fall to the ground, and new leaves quickly reemerge (Song et al., 2017). After this cycle, all the blades are replaced every two years in the springtime (Kleinhenz and Midmore, 2001; Mei et al., 2020). At the study site, the Moso bamboo forests were unevenly aged with both on-year and off-year characteristics. The recruitment of Moso bamboo shoots is high during odd-numbered years (on-year, e.g., 2017, 2019, and 2021) and low during even-numbered years (off-year, e.g., 2018, 2020, and 2022). This study included three age categories of culms: newly sprouted culms less than 1-year-old (the leaf formation was completed in June 2021), 2 or 4 years old (the leaf formation was completed in June 2020), and 6 or 8 years old (the leaf formation was completed in June 2020), referred to as young, mature, and old, respectively. The age of the selected culms was determined according to Mei et al. (2020). The crowns of the selected bamboo culms were intact, unbroken, and unaffected by visible diseases or pests. Thirty culms were selected as the sample plants (ten for each age category).

### 2.2 Measurements of leaf isoprene emission and photosynthetic characteristics

Several observation shelves were constructed with walkways to reach the crown of the bamboo leaves measured in situ. Using a portable photosynthetic system with a leaf chamber of 2 cm^2^, the photosynthetic parameters of Moso bamboo leaves were measured in the field (Li-6800; LI-COR Biosciences, Lincoln, BE, USA). Measurements from selected leaves were obtained on sunny days with daytime between 8:30 and 16:30 h. The analyzer was modified to sample isoprene by diverting the air at the outlet of the leaf chamber through a three-way valve (Pollastri et al., 2019; Riches et al., 2020), enabling a portion of the airflow to be drawn through a thermal desorption tube *via* an air sampling pump (produced by Beijing Municipal Institute of Labor Protection, China). The adsorption tube (Markes International, Ltd., Llantrisant, UK) filled with Tenax TA, Carbograph TD, and Carboxen 1003 adsorbents can absorb isoprene onto the filler because of its superior thermal stability and performance (Dettmer and Engewald, 2002). At the entrance to the leaf chamber, the air was filtered using an active carbon scrubber to remove atmospheric O_3_ and volatile organic compounds. Photosynthetic and isoprene emissions were measured with an airflow rate of 600 μmol s^-1^, and the CO_2_ concentration and relative humidity of the chamber were set as 400 µmol mol^-1^ and 60%, respectively. The light intensity and leaf temperature were set differently according to the experiment. The measured leaf was then enclosed in a chamber; isoprene was sampled after the photosynthesis reached a stable state (usually 10 to 20 min), and the photosynthetic parameters were synchronously recorded. Isoprene was sampled at a flow rate of 150 mL min^-1^ for 10 min. On each sampling day, one empty leaf chamber served as a blank sample that was analyzed using the same procedure. Finally, net isoprene emissions were calculated by subtracting the real samples from the blank samples. Measurements of leaf isoprene emission and photosynthetic characteristics were carried out on 30 sample culms.

#### 2.2.1 Seasonal variations

**Figure 1:**
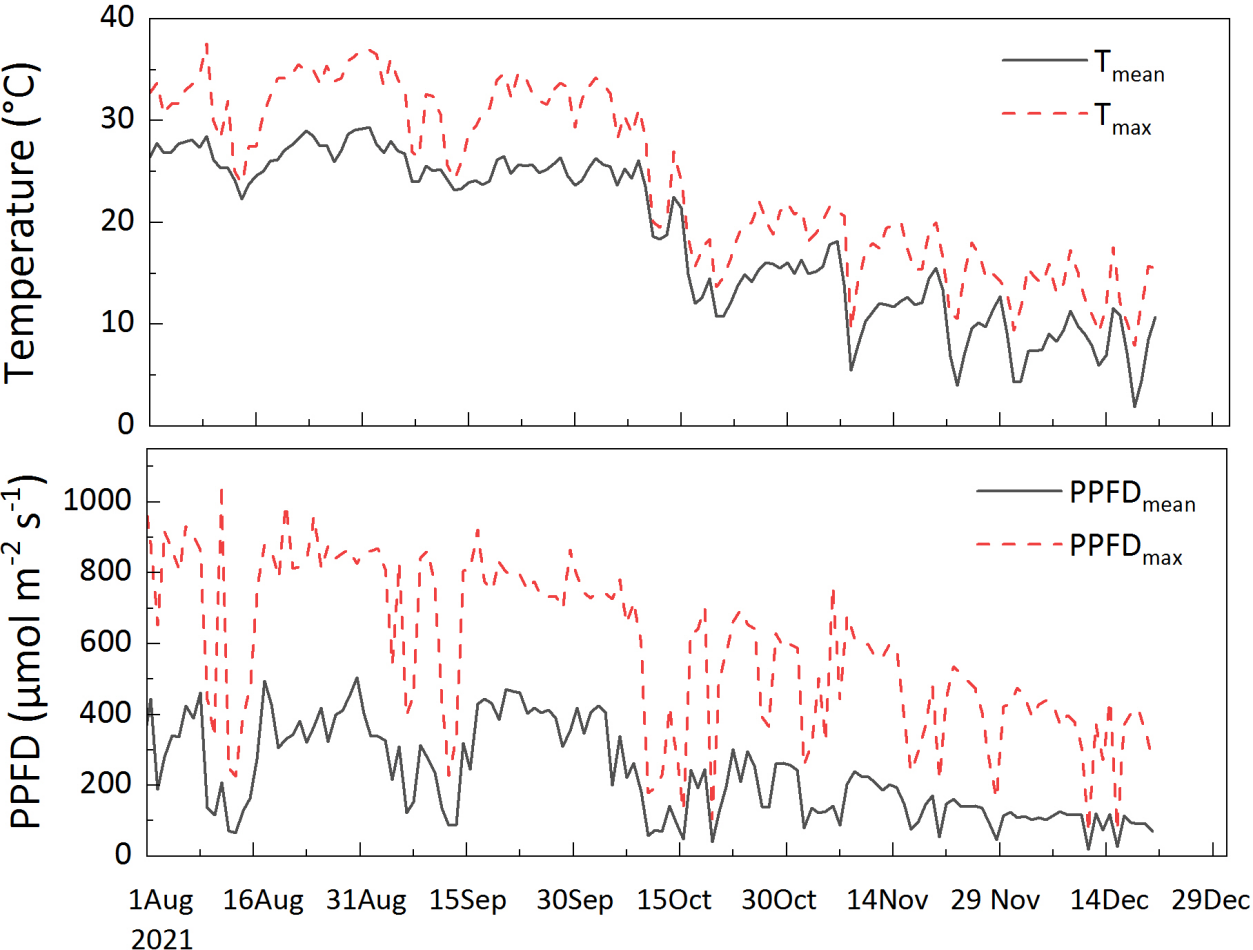
Diurnal (6:00-18:00) mean temperature (T_mean_), maximum temperature (T_max_), diurnal mean temperature (PPFD_mean_) and maximum photosynthetic photon flux density (PPFD_max_) during the experiment. The meteorological data were collected from a micrometeorological station in an open foothills area. The direct distance to the study plots was approximately 200 m. The station monitored radiation (MJ m^-2^; LI200X, Campbell, USA), and air temperature (Campbell, USA).

Seasonal variations were measured from summer to winter in 2021; no measurements were carried out in spring because it is the period of leaf change under an unstable physiological state. Data were collected in August (sampling dates: August 6–10 and 17–20, 104 leaves were measured), representing summer; September (sampling dates: September 7–10 and 17–20, 44 leaves were measured), October (sampling dates: October 2–5, 54 leaves were measured), and November (sampling dates: November 9–11, 52 leaves were measured), representing autumn; and December (sampling dates: December 2–8 and 21–22, 40 leaves were measured), representing winter. All measurements were performed under a photosynthetic photon flux density (PPFD) of 1000 μ mol m^-2^ s^-1^. The leaf temperature was set differently according to the ambient temperature: 30°C for August, September, and October; 15°C for November; and 10°C for December. The ambient temperature and light conditions monitored during the experiment are shown in **Figure 1**.

#### 2.2.2 Emission responses to light

The light response experiments for Moso bamboo leaves under constant leaf temperature (30°C) were evaluated on intact branches during September 2021. A thermal desorption tube sample was taken for each light intensity at PPFD values of 0, 100, 250, 500, 1000, and 2000 µmol m^-2^ s^-1^. Blank tubes were also collected without a leaf in the chamber when PPFD was 0 µmol m^-2^s^-1^. Additional details can be found in Jardine et al. (2016). Fourteen temperature response curves were measured, with four to six curves for each age category.

#### 2.2.3 Emission responses to temperature

The temperature response experiments for Moso bamboo leaves under constant PPFD (1000 μmol m^-2^ s^-1^) were evaluated in the field during September 2021 and November 2021, respectively. We performed two temperature response experiments to cover a wide temperature range (10–42°C). In September, we performed a high-temperature response experiment (27–42°C) because the ambient temperature was high, and the cuvette temperature was set to 27, 30, 33, 33, 36, 39, and 42°C in turn. While in November, we carried out a low-temperature response experiment (10–30°C) because the ambient temperature was relatively low, and the cuvette temperature was set to 10, 15, 20, 25, and 30°C in turn. After establishing the stable state of measurement, a thermal desorption tube sample was taken for each temperature. Blank tubes were also collected without a leaf in the chamber at an initial temperature of 27 or 10°C. Twenty-six temperature response curves were measured, with three to six curves for each age category.

### 2.3 Isoprene analysis and calculation of isoprene emission rates

After the samples were collected in the field, the adsorption tubes were sent back to the laboratory and stored in a refrigerator at 4°C for analysis within 7 days *via* thermal desorption-gas chromatography-mass spectrometry (TD-GC-MS, TD-20, GC-MS-QP 2010 SE; Shimadzu, Kyoto, Japan). A capillary column DB-624 (length: 30m, inner diameter: 0.25mm, film thickness: 0.14μm) was used. For desorption, the initial temperature of TD was set to -10°C, and the desorption time was 5 min for the first tube and 5 min for the cold trap hold. The split ratio was set at 1:1. The column temperature procedure was as follows: initial temperature of 30°C for 3.2 min, then increased to 50°C at 2°C min^-1^, and finally increased to 200°C at 8°C min^-1^ for 3 min. In this experiment, helium gas was used as the carrier gas, and the flow rates for desorption and gas chromatography-mass spectrometry (GC-MS) measurement were 30 mL min^-1^ and 1.2 mL min^-1^, respectively. MS analysis utilized 70 eV electron impact (EI) ionization and a mass scan range of 45–270 m/z. The transfer line temperature was 250°C, whereas the ion source temperature was 200°C (Yuan et al., 2020).

The concentration of isoprene was determined by fitting the data to a calibration curve generated from a standard of isoprene gas (99.9%; Ionicon Analytik GmbH, Innsbruck, Austria). The leaf isoprene emission rate (Iso, nmol m^-2^s^-1^) was calculated using Eq. 1 (Rodrigues et al., 2020):


(1) $$Iso=\frac{PA67\times Cal\times F\times 10^{-6}}{\;Area\;\times \;V}\;\;\;\;\;\;\;\;\;\;\;\;\;\;\;\;\;\;\;\;\;\;\;\;\;\;\;\;\;\;\;\;\;\;\;\;\;$$

where PA67 is the GC-MS peak area at the retention time for isoprene (ion counts of m/z 67 ×min), Cal is the calibration factor established for isoprene (10^-6^ nL isoprene/peak area), F is the air flow rate into the leaf chamber (600 mol s^-1^), and 10^-6^ is the conversion factor from mol to moles. The area is the leaf area contained within the 0.002 m^2^ chamber, and V is the total air volume that went through the adsorption tube (1.5 L).

Carbon loss from photosynthesis due to isoprene emissions was determined by the following formula:


(2) $$C–loss=\frac{6\;Iso}{\;Pn}\;\times 100\;\;\;\;\;\;\;\;\;\;\;\;\;\;\;\;\;\;\;\;\;\;\;\;\;\;\;\;\;\;\;\;\;\;\;\;\;\;\;\;\;\;$$

where both Pn and Iso are in the same molar unit, and the isoprene (C5 compound) emission rate is multiplied by six owing to the release of an extra CO_2_ molecule when 1-deoxyxylulose 5-phosphate is generated from glyceraldehyde phosphate and pyruvate (Sun et al., 2020).

### 2.4 Modeling the temperature and light responses of isoprene

The G93 algorithm (Guenther et al., 1993) is a commonly used model for calculating isoprene emission fluxes from plant leaves (Guenther et al., 2012; Sun et al., 2012; Lyu et al., 2021) and has been a successful fit for Moso bamboo (Okumura et al., 2018; Chang et al., 2019; Oku et al., 2021). The model G-93 estimates Iso as follows:


(3) $$\;Iso=\;Es\cdot C_{L}\cdot C_{T}\;\;\;\;\;\;\;\;\;\;\;\;\;\;\;\;\;\;\;\;\;\;\;\;\;\;\;\;\;\;\;\;\;\;\;\;\;\;\;\;\;\;\;\;\;\;\;$$

where Iso is the actual emission rate measured under different light and temperature, and Es is the emission rate under standard conditions (PPFD=1000 mol m^-2^s^-1^ and leaf temperature = 30°C), which is named the basal emission factor or emission capacity. C_L_ represents the light correction factor, and C_T_ is the leaf temperature correction factor. C_L_ is defined as follows:


(4) $$C_{L}=\frac{\alpha C_{L1}L}{\sqrt{1+\alpha^{2}L^{2}}}\;\;\;\;\;\;\;\;\;\;\;\;\;\;\;\;\;\;\;\;\;\;\;\;\;\;\;\;\;\;\;\;\;\;\;\;\;\;\;\;\;\;\;\;\;\;\;\;\;$$

where α and C_L_ are the empirical coefficients related to the light response (α=0.0027; C_L_=1.066). C_T_ is defined as follows:


(5) $$C_{T}=\frac{exp\frac{C_{T1}\left(T-Ts\right)}{RTsT}}{1+exp\frac{C_{T2}\left(T-T_{M}\right)}{RTsT}}$$

where R is the gas constant (8.314 J K^−1^ mol^−1^), Ts is the leaf temperature under standard conditions (30°C or 303K), T is the leaf temperature (K) at the time of sampling, C_T1_ and C_T2_ are the empirical coefficients related to leaf temperature, and T_M_ is an empirical coefficient related to the temperature of maximum isoprene emission. The values (C_T1 =_ 95000 J mol^-1^, C_T2 =_ 230000 J mol^-1^, and T_M_ = 314 K) were used in the original G93 algorithm (Guenther et al., 1993).

### 2.5 Assays of leaf morphology

The leaves of 30 sample plants (10 for each age group) were sampled to analyze leaf structural characteristics. Three samples were collected from each tree, and each sample comprised 50 randomly collected leaves. The fresh leaf mass was weighed, the leaf area was scanned, leaf thickness was measured, and leaves were further dried at 80°C for 48 h before calculating the dry mass of the leaves. The measurement details of leaf structural characteristics can be found in Yuan et al. (2020).

### 2.6 Statistical analysis

For the statistical analysis of age effects on the parameters of leaf morphology and characteristics of photosynthesis and isoprene emission addressed in this study, the data were compared by one-way ANOVA followed by Duncan’s multiple range test (P< 0.05). All statistical analyses were performed with SPSS 26 software (Statistical Product and Service Solutions, IBM, USA). Figures were drawn using Origin 2022b software (Origin Lab, USA).

## 3 Results

### 3.1 Leaf morphology of Moso bamboo with different culm ages

The foliage of mature and old trees had greater leaf area, leaf thickness, dry mass per unit area (M_A_), and dry-to-fresh mass ratio (DF) than young trees. In contrast, the leaf morphological traits of mature and old plants were similar (**Table 1**). Mature and old plants exhibited more developed photosynthetic mesophyll, but had lower water content. The leaf area, leaf thickness, and dry M_A_ of mature and old plants were 1.47 and 1.48, 1.33 and 1.44, and 1.28 and 1.34 times higher than those of young plants, respectively. This result indicates that the increase in dry M_A_ is correlated with the increase in leaf thickness.

**Table 1:** Mean ( ± SD) values of leaf morphological traits in young, mature, and old culms of Moso bamboo.

Traits	Young	Mature	Old
Leaf area (cm^2^)	5.95 ± 0.38 b	8.75 ± 0.75 a	8.81 ± 0.42 a
Leaf thickness (mm)	0.09 ± 0.01 b	0.12 ± 0.01 a	0.13 ± 0.01 a
Dry mass per unit area (M_A_, g m^-2^)	48.34 ± 2.30 b	61.64 ± 1.58 a	64.60 ± 2.29 a
Dry to fresh mass ratio (DF, g g^-1^)	0.50 ± 0.01 b	0.54 ± 0.00 a	0.53 ± 0.01 a

Data are mean ± SD of 10 independent bamboo culms, and different letters indicate statistically significant differences at P< 0.05.

### 3.2 Isoprene emission and photosynthesis in Moso bamboo with different culm age

**Figure 2:**
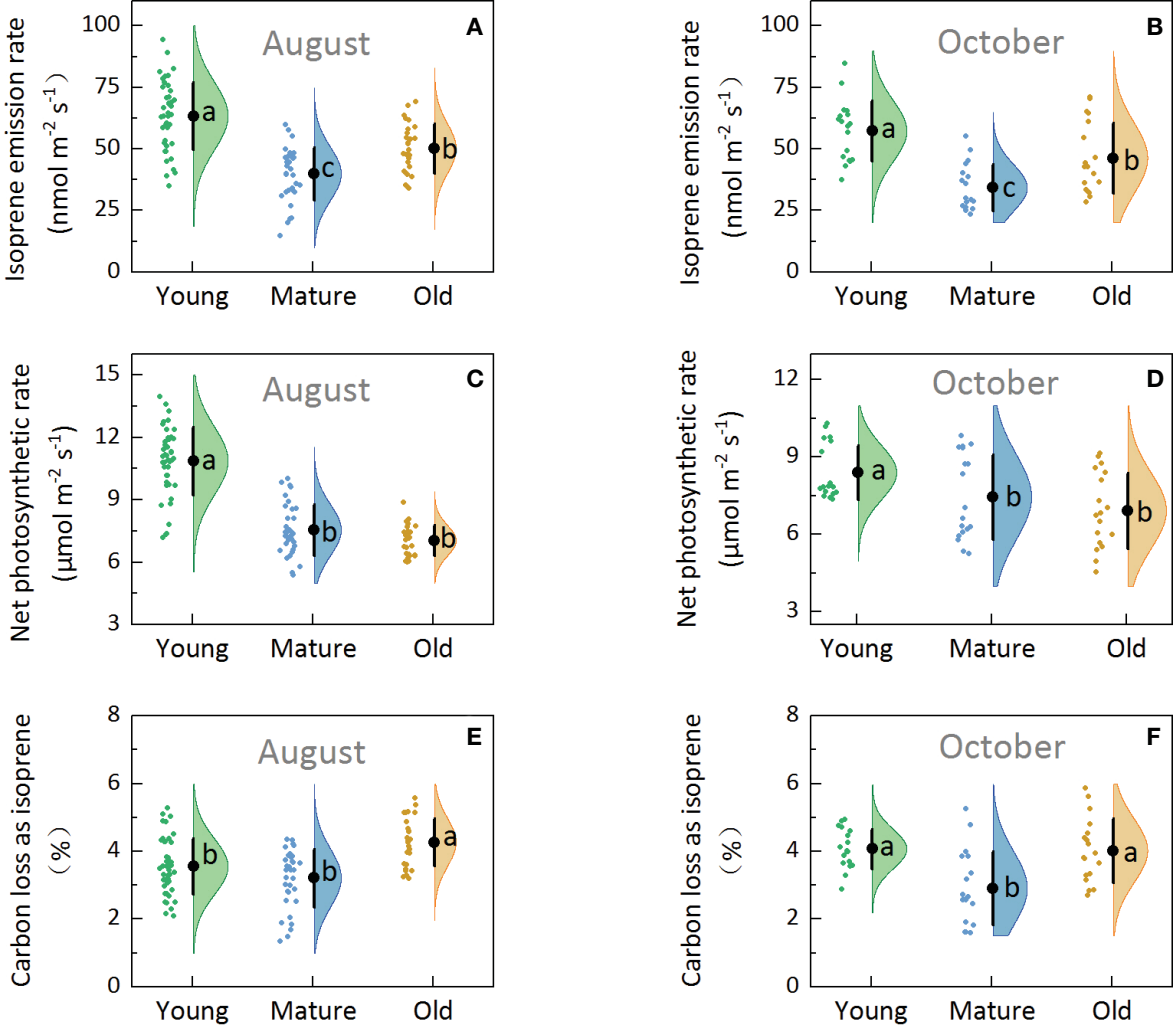
Rain cloud plot of isoprene emission and photosynthetic parameters with isoprene emission rate **(A, B)**, net photosynthetic rate **(C, D)**, percentage of carbon loss as isoprene **(E, F)** of Moso bamboo in August and October. The “cloud” represents the data distribution, and the “rain” represents the jittered raw data. The black point represents the mean distribution values, and the black line represents the standard deviations of the distributions. Lowercase letters indicate significant differences among young, mature, and old culms (P< 0.05).

Isoprene emission capacity (basal emission factor, Es), which was determined under standard conditions (30°C and 1000 µmol m^-2^ s^-1^ of PPFD), obtained in August (**Figure 2A**) and October (**Figure 2B**) showed that the Es was the highest in young (57.27–63.38 nmol m^-2^ s^-1^), followed by old (46.26–50.15 nmol m^-2^ s^-1^), and then mature plants (34.20–39.91 nmol m^-2^ s^-1^). The average Es of old plants was 30.09% higher than that of mature plants. Additionally, one-way ANOVA test results showed significant differences among the young, mature, and old groups (P<0.05), indicating that the Es of Moso bamboo was affected by culm age.

Net photosynthesis rate (Pn) was the highest in young (8.40–10.87 µmol m^-2^ s^-1^), followed by mature (7.45–7.55 µmol m^-2^ s^-1^) and old plants (6.92–7.04 µmol m^-2^ s^-1^) (**Figures 2C, D**), indicating that Pn decreased with the increase in culm age. One-way ANOVA revealed significant differences between young and mature and between young and old groups (P<0.05); however, no significant differences were observed between the mature and old groups (P>0.05).

Under standard conditions (leaf temperature: 30°C and light intensity: 1000 µmol m^-2^ s^-1^), the percentage of carbon loss from photosynthesis as isoprene emission (C-loss) was 2.75–4.26% (**Figures 2E, F**) in Moso bamboo. At any rate, mature plants showed the lowest C-loss, and the old plants showed significantly higher C-loss than the mature plants (P<0.05). The average C-loss of leaves was 3.81, 3.06, and 4.14 for young, mature, and old plants, respectively, and the C-loss of old plants was 35% higher than that of mature plants, indicating that the old bamboo paid the highest environmental cost.

### 3.3 Seasonal variations in isoprene emission and photosynthesis

**Figure 3:**
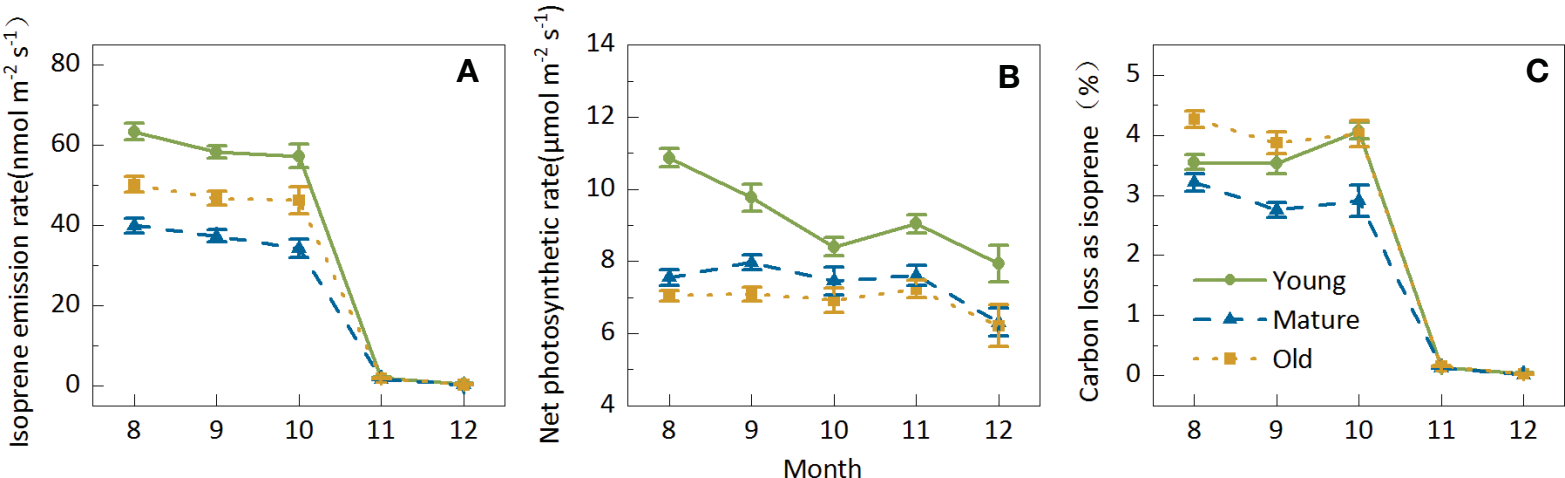
Seasonal variations in isoprene emission **(A)** net photosynthetic rate **(B)** and carbon loss as isoprene **(C)** of Moso bamboo and photosynthesis of Moso bamboo. Data are mean ± SE of 10–43 leaves from 267 samples for isoprene tests. During the measurements, leaf temperature was set to 30°C in August, September, and October; 15°C in November; and 10°C in December; ambient CO2 concentration was set to 400 mmol mol-1, and photosynthetic photon flux density (PPFD) was 1000 mmol m-2s-1.

The Iso from Moso bamboo leaves decreased with seasonal variations; however, the degree of decrease differed in different months. Iso slightly decreased by 10.24% from August to October; however, it sharply decreased by more than 96% to 1.83 nmol m^-2^ s^-1^ from August to October, and it further decreased to 0.31 nmol m^-2^ s^-1^ in December (**Figure 3A**). Nevertheless, the seasonal variation in Pn was not highly sensitive, ranging between 6.82 and 8.49 μmol m^-2^ s^-1^, and decreased by only 19.62% from August to December (**Figure 3B**). Owing to the small amplitude in Pn and the sharp decline in Iso, the seasonal variations in C-loss were similar to those in isoprene emission patterns. The average C-loss was 3.58% in August, September, and October and 0.14% and 0.03% in November and December, respectively (**Figure 3C**), indicating that C-loss was negligible in cold weather.

The seasonal patterns for the young, mature, and old groups were similar. In general, both Iso and Pn were the highest in the young group; however, the Iso of old plants was higher than that of mature plants, whereas Pn was higher in mature plants than in old plants. C-loss was the highest in old plants and the lowest in mature plants, indicating that mature plants consumed the lowest carbon cost, which may be conducive to biomass accumulation. Although the isoprene emission from Moso bamboo showed apparent seasonal variations, it showed a consistent age effect in different seasons, indicating that the age effect of isoprene emission characteristics was generally stable.

### 3.4 Light response of isoprene emission and photosynthesis

**Figure 4:**
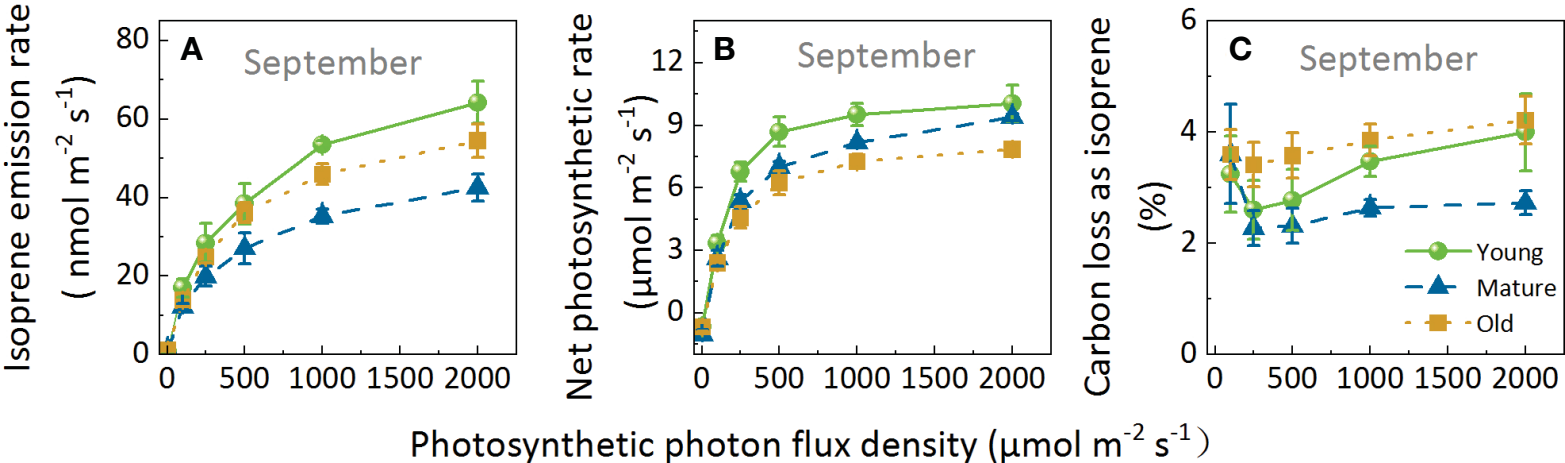
Isoprene emission rate **(A)**, net photosynthetic rate **(B)**, and percentage of carbon loss as isoprene **(C)** of Moso bamboo leaves in response to light intensity. During the measurements for light response, leaf temperature was set to 30°C, and ambient CO_2_ concentration to 400 μ mol mol^-1^. Data are means ( ± SE), n=4–6.

Regarding Iso and Pn, the leaves of different culm ages exhibit a similar pattern in response to light; both parameters increased with increasing light intensity (**Figures 4A, B**). However, the slope of the curve of Iso was lower but had a higher light saturation point than Pn. Pn reached the light saturation point at PPFD of 1000 μmol m^-2^ s^-1^, whereas Iso was not saturated at this point and continued to rise with increasing PPFD. Although the response patterns of the different groups were similar, the response values differed in the following order: Iso: young > old > mature; and Pn: young > mature > old.

The fluctuation range of C-loss was 2.28–4.21% (**Figure 4C**). Overall, C-loss initially decreased and then increased, and the lowest peak appeared at a PPFD of 250 μmolm^-2^s^-1^ for all groups: 2.60, 2.28, and 3.14% for the young, mature, and old groups, respectively. Subsequently, C-loss increased with increasing PPFD; when PPFD increased to 2000 μmol m^-2^ s^-1^, the C-loss for the young, mature, and old groups increased by 53.60%, 19.57%, and 23.30%, respectively, indicating that Iso was more stimulated in young plants than in mature and old ones under high light intensity. Moreover, C-loss was the highest in the old group, followed by young, then mature groups. At a PPFD of 2000μmol m^-2^ s^-1^, C-loss in the mature group was 31.82% and 64.73% lower than that in the young and old groups, respectively. The above results indicated that although the values were different under different light intensities, the response patterns of different ages to light are similar, and light intensity does not change the age effect.

### 3.5 Temperature response of isoprene emission and photosynthesis

We performed two temperature response experiments, including a temperature range of 27–42°C (**Figures 5A–C**) and 10–30°C (**Figures 5D–F**) in September and November, respectively. Iso increased with increasing temperature and then decreased at temperatures >39°C. On the other hand, Pn decreased when temperatures were >30°C in September (**Figure 5B**) and >25°C in November. Moreover, Pn was less sensitive than Iso in response to temperature. For example, in a temperature range of 10 to 30°C, the coefficient of variation of Pn was 0.13, while the coefficient of variation of Iso was up to 0.96 (**Figures 5D, E**). Similarly, C-loss varied greatly and increased with increasing temperature; When the temperature was below 20°C, C-loss was less than 0.5%, and when the temperature was 42°C, the C-loss exceeded 25% (**Figures 5C, F**).

**Figure 5:**
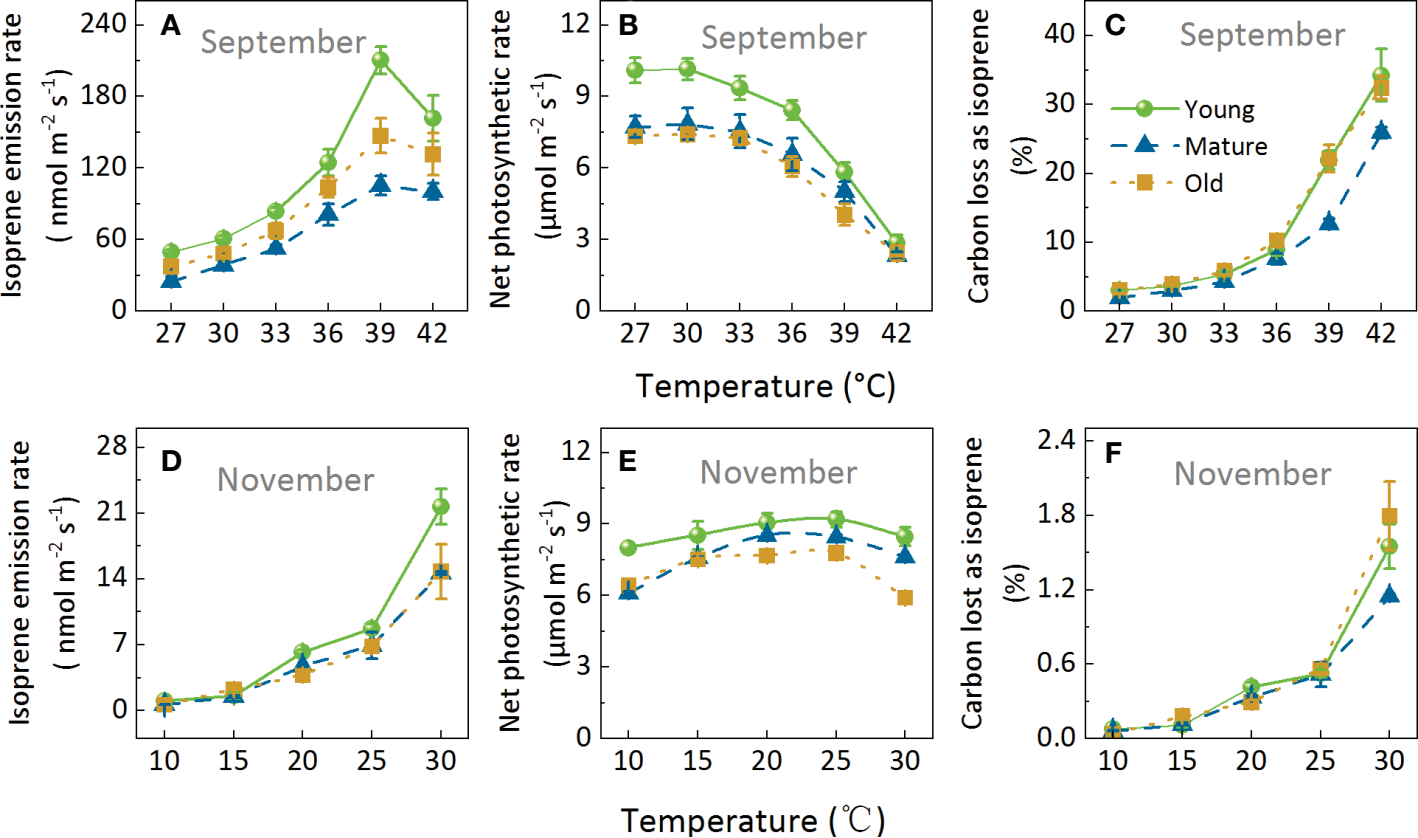
Effects of increasing temperature on isoprene emission rate **(A, D)**, net photosynthetic rates **(B, E)**, and carbon lost as isoprene **(C, F)**. Measurements were taken in September **(A–C)** and November **(D–F)**. During the measurements, ambient CO_2_ concentration was set to 400 μmol mol^-1^, and photosynthetic photon flux density (PPFD) was 1000 μmol m^-2^s^-1^ Data are mean ± SE (n = 3–6).

The variation range of Iso differed among the different age groups, especially when the temperature increased from 36°C to 39°C (**Figure 5A**). Iso increased the most in the young group, followed by the old group and then the mature group, by 68.91, 41.65, and 29.80%, respectively. However, it was worth noting that Pn decreased the most in the young group (**Figure 5B**) and finally led to very high C-loss (**Figure 5C**). The above results showed that Iso, Pn, and C-loss were strongly influenced by temperature, and that different age groups responded differently to temperature, with high temperature having the most significant effect on young bamboo.

### 3.6 Modeling isoprene emission from bamboo leaves

**Figure 6:**
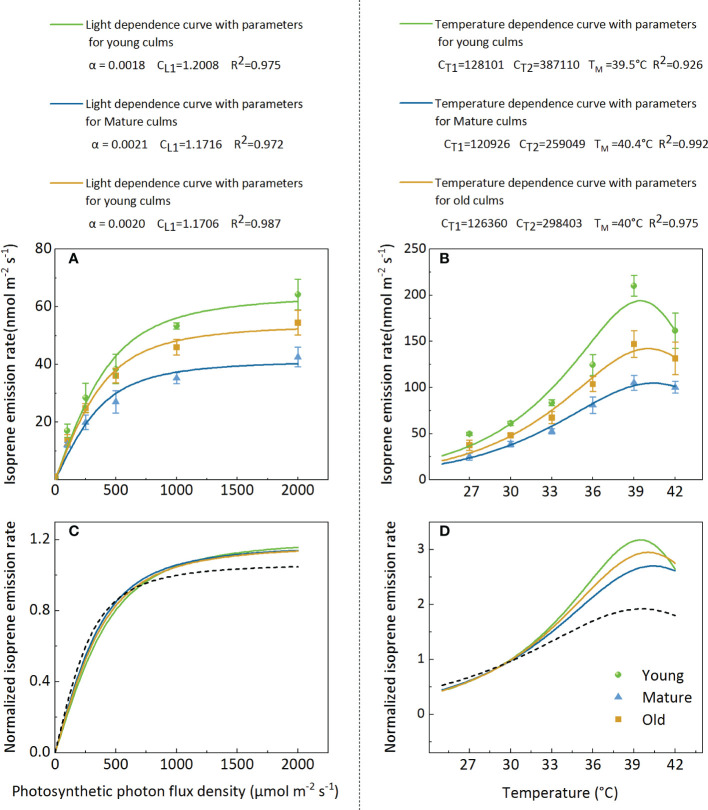
Representative light **(A, C)** and temperature **(B, D)** dependencies of isoprene emission rate **(A, B)** and normalized isoprene emission rate **(C, D)** for leaves in young, mature, and old bamboos. Data in **(A, C)** were fitted using Eq.3 with the parameters defined as follows: Es-the isoprene emission capacity, α-the initial quantum yield of isoprene emission, and C_L1_-a scaling constant. Data in **(B, D)** were fitted by Eq.4, C_T1_ and C_T2_ are parameters which can be interpreted as activation and deactivation energy of isoprene emissions (in J mol^-1^), respectively; T_M_ presents the optimum temperature for isoprene emission.

The model fit indicates that attempts to model isoprene emissions using the G93 model (Guenther et al., 1993) were successful (**Figure 6**). However, the parameters (α, C_L1_, C_T1_, C_T2_, and T_M_) obtained from the best fit to the data from Moso bamboo were different from the original parameters of the algorithm G93 (**Figures 6A, B**). The isoprene emission rate data were normalized to more intuitively display the light and temperature functions of the different ages (**Figures 6C, D**). The results showed that the light function with the original parameters of the algorithm G93 underestimated isoprene emission at a high PPFD level (>750 µmol m^-2^s^-1^) for all ages (**Figure 6C**), and the curves of different groups almost coincided. For temperature function, the original parameters of the G93 algorithm underestimated isoprene emission at temperatures>30°C and overestimated it at temperatures<30°C. Notably, curves of the groups with different culm ages almost coincided when the temperature was<30°C but was clearly separated at temperatures >30°C, especially at 39°C (**Figure 6B**).

## 4 Discussion

### 4.1 Age effects on leaf isoprene emission

In this study, the photosynthesis and isoprene emissions of Moso bamboo leaves were affected by culm age under standard environmental conditions, different seasons, light response, and temperature response. The leaf photosynthetic capacity decreased with increasing culm age; however, the isoprene emission rate followed the order of young >old >and mature plants. The effects of culm age on photosynthesis observed in our study are consistent with those of previous studies (Kleinhenz and Midmore, 2001; Peng et al., 2021). As expected, young plants had the highest isoprene emission rate because the biosynthesis of isoprene is based on a photosynthetically fixed carbon (Niinemets et al., 2002; Jardine et al., 2014; de Souza et al., 2018). Young culms had the highest photosynthetic rate and isoprene emission, which may be also related to the younger leaf age and different morphology (Niinemets et al., 2005). Notably, the isoprene emission rate of old plants was higher than that of mature plants; however, the opposite trend was observed for Pn. Carbon allocation, a critical physiologic mechanism in which photosynthates are redistributed between respiration and biomass synthesis, temporary and permanent tissues, and aboveground and belowground components, may be responsible for this effect. According to the optimum equilibrium theory, plants distribute their resources among their many organs to achieve maximum fitness as a whole (Bloom et al., 1985; Poorter and Nagel, 2000; Jansson et al., 2021). Underground rhizomes connect the aboveground culms of Moso bamboo of different ages. Some studies have suggested that mature bamboo may provide water, nutrients, and carbohydrates to bamboo shoots and juvenile bamboo (Song et al., 2016; Sun et al., 2019; Mei et al., 2020; Wang et al., 2020). Carbohydrate dynamics and photosynthesis in Moso bamboo have been studied by Wang et al. (2020). Mature bamboo (at ages 2, 3, and 4) has been shown to have a functioning photosynthesis system and the ability to produce carbohydrates; nearly all mass fractions of non-structural carbohydrates (NSCs) are low in mature bamboo owing to their transfer into structural carbohydrates (SCs) and use in other biosynthetic and metabolic pathways. However, old bamboos have a low transport capacity of sucrose (Wang et al., 2020). Based on the above discussion and our results, we speculate that although the photosynthetic capacity of mature plants is high, the photosynthate transfers rapidly, resulting in a low substrate level for isoprene synthesis. However, although the photosynthetic capacity of old plants is slightly lower than that of mature plants, the low transport capacity of old plants leads to photosynthate accumulation in the leaves, which may result in higher substrate levels and isoprene emission rates. In addition, the carbon distribution of photosynthates between isoprene synthesis and primary metabolism may also account for the differences in isoprene emission rates at different culm ages (de Souza et al., 2018; Huang et al., 2019; Monson et al., 2021). Besides isoprene, we also detected other volatile compounds, and the data showed that isoprene was the dominant substance released from all the measured leaves. The other volatiles are less than 2% in total, and no green leaf volatiles (GLVs) had been detected, which may be related to the fact that the bamboo leaves we measured are mature but have not yet entered the senescence stage.

Irregularly aged stand structures characterize Moso bamboo forests because they are made up of culms of varying ages. Older culms (over 4–5 years old) should be harvested to sustain the stand productivity (Yen and Lee, 2011; Zhou et al., 2011; Yen, 2017); therefore, artificially managed bamboo forests are usually composed of 1–5 years old culms. However, a drop in the economic benefits of bamboo has recently been observed owing to the falling price of bamboo and rising labor expenses. Many bamboo forests, especially those of higher elevations or more complicated terrains, have been abandoned as their owners no longer care to maintain them (Suzuki and Nakagoshi, 2007; Yin et al., 2019). Our results showed that the Iso of old bamboo is higher than that of mature bamboo, indicating that if Moso bamboo forests are abandoned and the old culms are not harvested, the potential negative impact of bamboo forests on the environment may increase. This strategy of harvesting old plants coincided with the perspective of sustainable management of Moso bamboo forests. Nevertheless, the ecological or economic benefits of the old plants should also be considered when planning a bamboo forest management strategy.

### 4.2 Seasonal variations

Environmental conditions generally exhibit apparent seasonal changes, with higher temperatures and light in summer and lower temperatures and light in winter. Inherent leaf capacity to synthesize isoprene may depend on the availability of the isoprene precursor and isoprene synthase activity, which are correlated with seasonal variations in isoprene emissions and are species-specific (Kuhn et al., 2004; Mu et al., 2022). In this study, the isoprene emission rate was the highest in summer and the lowest in winter, which can be mainly attributed to environmental drivers such as temperature and light. We observed that isoprene levels sharply decreased from October to November owing to two cold snap events occurred during measurements. Notably, the primary plant physiology reflected by net photosynthesis was not significantly affected, indicating that the availability of the isoprene precursor substrate, which mainly originates from photosynthesis, did not considerably change. However, isoprene synthase activity strongly depends on the temperature (Monson et al., 1992; Rasulov et al., 2010; Alves et al., 2014; Taylor et al., 2019). In addition, leaf phenology and physiology, nutrient and water availability, and even pre-measurement growth circumstances may all have a role in seasonal fluctuations (Sharkey and Loreto, 1993; Alves et al., 2014; Mu et al., 2022).

### 4.3 Response to light and temperature

Temperature and light conditions greatly affect the growth of plants. In addition to their regulatory roles in plant growth and productivity, light and temperature affect isoprene synthesis and emission. This study validated the light dependency of isoprene production and emission, as reported in earlier studies, with the response of isoprene emission and photosynthesis to changing light levels approximating a rectangular hyperbola (Lerdau and Keller, 1997; Alves et al., 2014; Oku et al., 2021). This can be owed to the energy and reductive equivalents for the *de novo* synthesis of isoprene that are provided by a photosynthetic electron transport (Wang et al., 2022). Results indicated that although photosynthesis saturates at 1000 mol m^-2^s^-1^ of PPFD, isoprene emission does not saturate until up to 2000 μmol m^-2^ s^-1^ of PPFD, indicating that the two processes are not completely coupled. This effect has also been observed in subtropical and tropical species (Lerdau and Keller, 1997; Alves et al., 2014; Jardine et al., 2016; Garcia et al., 2019); however, the underlying cause of this effect is not fully understood. Isoprene emission may reach its saturation at higher irradiance than photosynthesis for various reasons, one of which is that photosynthesis may not be able to fully utilize all the ATP or NADPH generated by electron transport under the conditions of intense light, leaving an excess available for isoprene production, as proposed by Niinemets et al. (2002). Moreover, in this study, temperature response curves showed that isoprene was uncoupled from photosynthesis when the leaf temperature was higher than the optimal temperature for photosynthesis, which is consistent with previous studies (Sharkey and Loreto, 1993; Sharkey et al., 1996; Pollastri et al., 2019). This classic uncoupling is related to the use of alternative carbon sources (de Souza et al., 2018). Isoprene emission, which significantly increased at high temperatures, may be an important reason for the high flux of isoprene observed over land during hot seasons (Wang et al., 2013; Hong et al., 2019). Moreover, based on the isoprene response pattern to temperature, we predict that global warming will increase isoprene emission from plant.

Isoprene emissions from plants help them endure abiotic stresses like heat, drought, and cellular oxidative damage (Monson et al., 2021). This seems to be a crucial feature that shields photosynthesis mechanism of plants from the adverse impact of climate (Loreto et al., 2006; Sharkey et al., 2008). A modest fraction of the net photosynthesis rate is contributed by the isoprene emissions of many plant species under ideal environmental conditions for photosynthesis (Okumura et al., 2008; de Souza et al., 2018). However, under stress conditions, such as high leaf temperatures, that reduce the net photosynthesis rate but increase isoprene emissions, these emissions can account for 10–50% of the net photosynthesis rate (Sharkey and Loreto, 1993; Harley et al., 1999; Jardine et al., 2014; Jansson et al., 2021). In this study, the effect of temperature on C-loss was considerably greater than that of light. At any light intensity, the value of C-loss was<5%, and the range of variation was narrow; however, it was considerably wide at different temperatures: when the leaf temperature was<15°C, the C-loss was<0.2%; when the leaf temperature was between 33°C and 36°C, the range of C-loss was 4.3–10.26%; and when the leaf temperature was 42°C, the C-loss was as high as 25.94–34.25%, indicating severe temperature stress. Thus, the release of isoprene should be considered when calculating the carbon budget.

Notably, different culm ages appear to exhibit different sensitivities to temperature; young plants were the most sensitive, especially under high-temperature conditions. When the temperature increased from 30°C to 39°C, young plants exhibited the largest reduction in Pn. A significant decrease in Pn indicates that carbon fixation is the most affected; however, a substantial increase in Iso indicates that the largest fixed carbon is consumed. This result is consistent with the observed leaf morphology; young leaves had the thinnest leaf area that stored the minimum NSCs, and the small thickness of leaves cause sensitivity to temperature. This explains why young leaves are more prone to curl in the summer (high temperature) season. Our study may also provide novel insights into the cause of the high death rate of young plants under high temperatures and drought events in China in 2013 (Pei and Shi, 2017).

### 4.4 Modeling isoprene emission from leaves should include culm age

The present study revealed that the basal isoprene emission factor (Es) varied with different culm ages, and the parameters (α, C_L1_, C_T1_, C_T2_, and T_M_) obtained from the best fit to the data from Moso bamboo were different from the original parameters of the algorithm G93. Therefore, optimized parameters should be adopted, and models should be established based on different culm ages to accurately estimate isoprene emissions from Moso bamboo forests. Notably, the maximum temperature for isoprene emission was different for plants with different culm ages, with 40.4, 40.0, and 39.5°C for the mature, old, and young plants, respectively, thereby providing further evidence for the temperature tolerance of culms of different ages.

## 5 Conclusions

This study quantified the effects of culm age on leaf isoprene emissions, synchronously monitored photosynthetic parameters, and showed that culm age significantly affected isoprene emission from the leaves. We recommend including the age factor in estimating the isoprene emission flux of Moso bamboo forests to improve the estimation accuracy. Moreover, because the Iso and C-loss of old bamboo were higher than those of mature bamboo, we suggest that attention should be paid to the management of bamboo age structure and timely felling of aged bamboo to reduce the environmental risk. Moreover, research covering a more extensive age range needs to be carried out to comprehensively understand the age effect.

## Data Availability

The original contributions presented in the study are included in the article/supplementary materials. Further inquiries can be directed to the corresponding author.
