# Corrigendum: Age effects of Moso bamboo on leaf isoprene emission characteristics

**DOI:** 10.3389/fpls.2024.1521031

**Published:** 2024-12-10

**Authors:** Yandong Song, Chunju Peng, Qinjiao Wu, Shijie Tao, Tingting Mei, Zhihong Sun, Zhaojiang Zuo, Chunyu Pan, Yufeng Zhou, Guomo Zhou

**Affiliations:** ^1^ State Key Laboratory of Subtropical Silviculture, Zhejiang A&F University, Hangzhou, China; ^2^ Lishui Academy of Agricultural and Forestry Sciences, Lishui, China; ^3^ Wenzhou Vocational College of Science and Technology, Wenzhou, China; ^4^ Key Laboratory of Carbon Cycling in Forest Ecosystems and Carbon Sequestration of Zhejiang Province, Zhejiang A&F University, Hangzhou, China; ^5^ College of Horticulture Science, Zhejiang A&F University, Hangzhou, China; ^6^ Zhejiang Provincial Key Laboratory of Forest Aromatic Plants-based Healthcare Functions, Zhejiang A&F University, Hangzhou, China; ^7^ Faculty of Forestry, University of British Columbia, Vancouver, BC, Canada

**Keywords:** Moso bamboo, isoprene, photosynthesis, light dependency, temperature dependency, G93 algorithm

In the published article, there was an error in the **Funding** statement.

“This study was funded by the Key Research and Development Program of Zhejiang Province (Grant No. C02005); Lishui Key Scientific and Technological Innovation Team (Grant No.2018cxtd02); the National Nature Science Foundation of China (Grant No. 2045210652, 32001102); Scientific Research Foundation of Zhejiang A∓F University (Grant No. 2020FR050); Scientific Research Foundation of Jiyang College of Zhejiang A&F University (Grant No. 05251700038); the Overseas Expertise Introduction Project for Discipline Innovation (111 Project D18008).”

The correct **Funding** statement appears below.

“This study was funded by the Key Research and Development Program of Zhejiang Province (Grant No. 2021C02005); Lishui Key Scientific and Technological Innovation Team (Grant No.2018cxtd02); the National Nature Science Foundation of China (Grant No. 32071731, 32001102); Scientific Research Foundation of Zhejiang A&F University (Grant No. 2020FR050); Scientific Research Foundation of Jiyang College of Zhejiang A&F University (Grant No. 05251700038); the Overseas Expertise Introduction Project for Discipline Innovation (111 Project D18008).”

The authors apologize for this error and state that this does not change the scientific conclusions of the article in any way. The original article has been updated.

